# Diagnosing Cervical Dysplasia Using Visual Inspection of the Cervix with Acetic Acid in a Woman in Rural Haiti

**DOI:** 10.3390/ijerph111212304

**Published:** 2014-11-28

**Authors:** Elizabeth Roger, Oguchi Nwosu

**Affiliations:** Emory University School of Medicine, Atlanta, GA 30322, USA; E-Mail: eroger@emory.edu

**Keywords:** cervical cancer, visual inspection of the cervix with acetic acid (VIA), cryotherapy, screen and treat, Haiti, mobile clinics

## Abstract

Cervical cancer remains a significant cause of morbidity and mortality for women in developing countries, despite the fact that inexpensive, simple and effective screening methods are available. Visual inspection of the cervix with acetic acid (VIA) can be used as part of a “screen and treat” program to identify precancerous lesions for cryotherapy treatment. This case report details how the VIA screening test was incorporated into the care of a patient presenting to a maternal health clinic in Thomonde, Haiti which was staffed by doctors and medical students from Emory University School of Medicine in collaboration with Haiti Medishare. As demonstrated here, the VIA test requires minimal materials, can be efficiently incorporated into a physical exams, provides immediate results, and is easily demonstrated to and performed by local healthcare providers. The straightforward and sensitive VIA technique is an ideal cervical cancer screening method for resource poor areas.

## 1. Introduction

Cervical cancer is the second most frequently diagnosed cancer in women worldwide. A disparate 88% of deaths from cervical cancer occur in developing countries, making it the most common cancer in the developing world [1]. Haiti has the highest incidence and mortality rates of cervical cancer in the Western hemisphere, and some sources estimate the highest incidence in the world (93.9 per 100,000) [2,3]. Their mortality rate, nearly 30 times the rate that occurs in United States, is due to lack of effective screening and treatment programs.

Exposure to human papillomavirus (HPV), along with synergistic risk factors including multiple sexual partners, early age of first intercourse, poverty, multi-parity, tobacco use, malnutrition, and poor genital hygiene increases a woman’s risk of progression to cervical cancer [4]. These factors initiate a long pre-cancerous stage during which screening, diagnosis, and intervention programs have been effective historically in decreasing cervical cancer mortality [5]. Efforts have been made to institute screening programs in resource-poor countries, although significant barriers exist. Health programs lack the funds for screening tests that utilize cytologic samples, the expertise to read and diagnose the samples, and the record and communication systems to track and inform women with abnormal test results [6]. Cytology laboratories are expensive to maintain and there are often delays before the results become available, leading to issues with follow up when transportation difficulties exist. Although vaccination offers a complementary preventative strategy for reducing cervical cancer mortality in women who have not yet acquired HPV, cost remains prohibitive to implementation of a thorough vaccination campaign in many resource poor settings [5].

**Figure 1:**
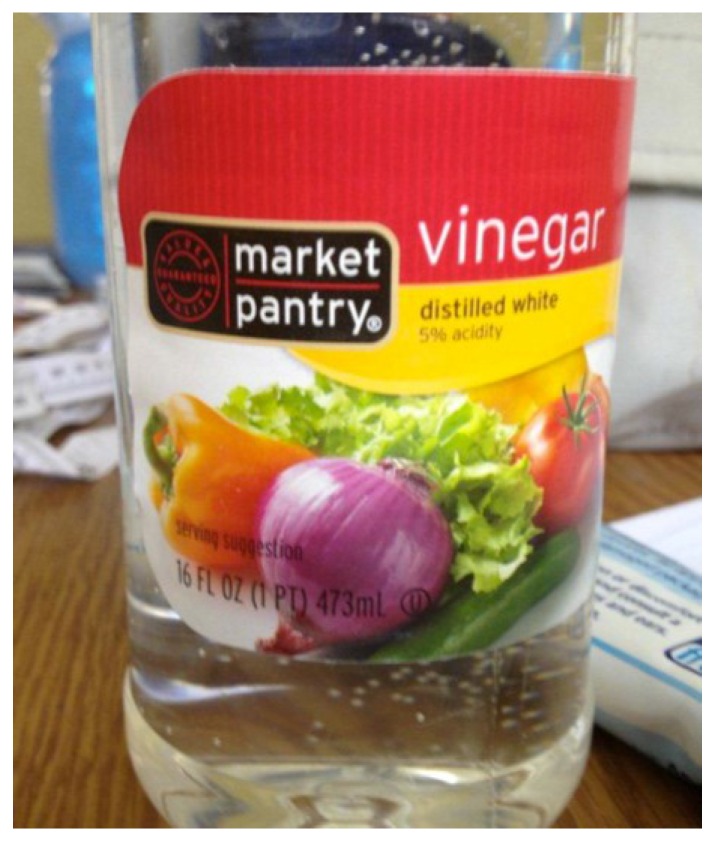
Inexpensive household vinegar used to perform the Visual Inspection with Acetic Acid (VIA) test.

Non-cytologic tests, such as visual inspection of the cervix with acetic acid (VIA) avoid reliance on expensive laboratory equipment and overcome other recognized barriers [7]. Common household vinegar applied on the cervix will cause areas of dysplasia to appear bright white (Figure 1). This screening test can be performed at the bedside by a range of trained providers including physicians, nurses and nurse-midwives, and has been shown to be safe and efficacious [8,9]. The ability to obtain instantaneous results allows for immediate treatment and reduces loss to follow-up. Evaluations of VIA in cross-sectional studies report sensitivities of 75% and 76.8% in detecting precancerous lesions using colposcopy with biopsy as a reference test [8,10,11]. Although Papanicolaou (Pap) screening has been shown to be more specific, using VIA first in a combined screening technique can increase overall sensitivity and specificity significantly [6,12,13]. Studies done on cost-effectiveness of screening find optimal benefit in offering VIA to women every five years from the ages of 30 to 45 and Pap smears after age 50 [14]. Screening with VIA has been shown to be effective in low resource settings at decreasing the prevalence of high-grade precursor lesions and the low cost and simplicity of the procedure affirm its aptitude as an initial screening tool [8,13].

In addition, the HPV DNA test, termed careHPV^®^ (Qiagen, Alameda, CA, USA) has been shown to be comparable to Pap tests and could be combined with VIA to screen for cervical cancer screening in resource poor settings [15]. Technicians can be trained quickly to perform the test and women can self-swab, eliminating the requirement for highly trained health professionals to perform examinations and reducing the need for expensive lab equipment [16,17].

Here we discuss a case of a 50 year old Haitian multiparous woman with 11 previous births who presented to a maternal health clinic in Thomonde, Haiti, staffed by physicians and medical students from Emory University School of Medicine as part of a collaboration with the Haiti Medishare program. The patient had multiple complaints due to a lack of previous healthcare. We will focus this report mainly on her gynecological symptoms and the subsequent clinical examination that was performed, including the application of the VIA screening technique.

## 2. Case Presentation

A 50 year old P11G11 female presented to a Maternal Health clinic near Thomonde, Haiti with vaginal complaints. The patient described a 7-year history of vaginal symptoms including white, copious, malodorous discharge accompanied by itching of the vulva and vaginal vault that began after the delivery of her last child. She denied dyspareunia, postcoital spotting and flank pain. Her 11 pregnancies were uncomplicated and all deliveries were vaginal. Her last menstrual period was within the last week, although her periods were becoming intermittent. Patient denied any history of STIs and had never received a Pap smear or speculum exam. She had not received regular gynecological care and was reporting these symptoms for the first time.

Apart from some knee pain and breast pain near the incision site of an earlier breast biopsy, she had no other complaints. She had not sought medical attention for any of these complaints for the past seven years. She was otherwise healthy. She was married with lifetime monogamy, and she denied smoking and alcohol use. She was taking no medications and had no history of allergic reactions to medications.

**Figure 2:**
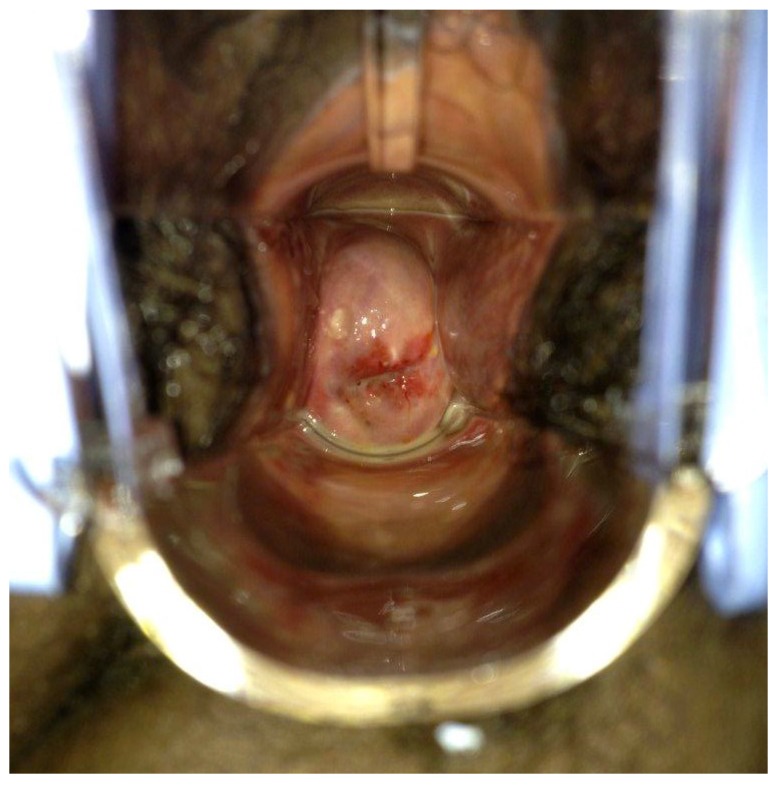
Speculum exam revealed erythematous, swollen cervix with white discharge and notable vascular changes surrounding the os.

On examination her blood pressure was 120/78 and heart rate was 82 beats per minute. The patient appeared well, attentive, and oriented. Her heart and lung exams were within normal limits. Three well-circumscribed, mobile, half-centimeter nodules were palpable in right breast. Abdominal exam revealed no palpable masses, ascites or costovertebral angle tenderness. Pelvic exam revealed normal external female genitalia. Speculum exam allowed the visualization of an erythematous, swollen cervix with copious white discharge from the cervical os and notable vascular changes surrounding the os (Figure 2) On manual exam the patient was noted to have cervical motion tenderness. Palpation revealed a firm cervix with rigid, gritty nodules at 12 o’clock. Ovaries and uterus were normal to palpation. Wet prep was negative for both clue cells and hyphae.

**Figure 3:**
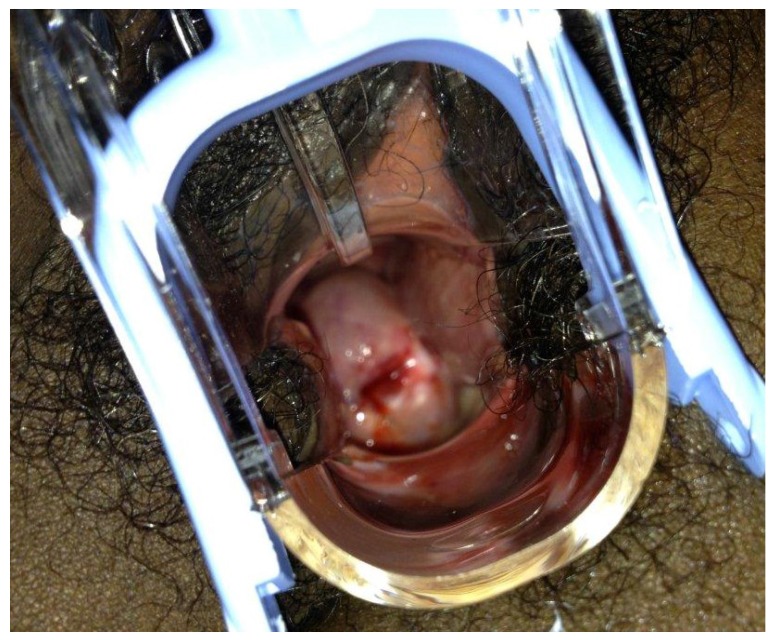
Visualization of cervix with acetic acid reveals white patches indicating cervical dysplasia.

Using cotton swabs, common household vinegar containing 5% acetic acid was applied copiously over the cervix. Within seconds, portions of the cervix appeared bright white, providing evidence of potential cervical dysplasia (Figure 3) Acetowhite change was noted at the 3, 5, and 6 o’clock positions on the cervical portio. The most significant white color change was a 3 mm lesion at 3 o’clock, approximately 2 mm from the external os. In addition, prominent and tortuous vessels were noted surrounding the os.

The patient was treated empirically with oral doxycycline and metronidazole to cover bacterial and protozoal causes of pelvic inflammatory disease and vaginal infections. She was referred to the hospital in Hinche for more thorough gynecological follow-up, including follow up for her breast complaints and appropriate subsequent treatment for the potential cervical dysplasia and/or cervical cancer observed during the VIA screening test.

## 3. Discussion

The case reported here demonstrates the ease with which the VIA screening method can be incorporated into a physical exam in resource-poor settings. The technique is quick to perform and utilizes accessible, low cost materials, making it an ideal option for screening in resource poor settings [18]. The simplicity of the screening test makes it possible to train health care professionals rapidly, and the procedure is safe and effective in identifying pre-cancerous lesions [8,9]. However, studies also draw attention to the disadvantages of VIA such as the confounding nature of cervicitis on VIA results and the variation of the test’s sensitivity and specificity which ranges from 41.4%‒93.9% and 74.2%‒93.8%, respectively, according to eleven cross sectional studies done in resource poor sites in India and Africa [11,19]. Although this variability in cited sensitivity and specificity exist, there is no doubt that this screening test removes the need for a fully staffed and equipped laboratory, and thus allows screening programs to reach more remote areas. It enables the clinician to deliver immediate results to patients who often face geographical and transportation obstacles that interfere with delivery of follow-up reports and appropriate care [6,7]. Despite its cost-effectiveness and ease of application, the VIA screening technique remains under-utilized. VIA is by no means a novel screening tool and knowledge of the effectiveness of the method has existed for many years. In particular, Surendra Shastri’s 12 year randomized controlled trial looking at the effectiveness of VIA in Mumbai, India, showed a significant reduction in cervical cancer mortality [20]. Additional studies in Thailand, rural China, Kenya, South Africa, Zimbabwe, the Philippines, Democratic Republic of the Congo, Pakistan, and Guatemala provide evidence of the cost effectiveness, ease of implementation, and reduction in morbidity and mortality that VIA screening provides [5,8,9,12,14,15,21]. However, VIA has yet to become a routine method of cervical cancer screening.

As evident in the high rate of cervical cancer incidence and mortality that remain in the developing world, “screen and treat” programs for cervical dysplasia are desperately needed [1]. The method of VIA offers a potential option that is cost-effective and can be combined with other screening and treatment procedures such as HPV DNA testing, PAP smears, and colposcopy [6,17]. Cryotherapy provides the option to immediately treat abnormal findings once they are observed and reduces those lost to follow up care. When this service is not available, as in the case presented here and many rural settings, there is the option to refer patients to sites where they can have a diagnostic or therapeutic procedure such as biopsy or cryotherapy. Combining preventive strategies, such as vaccination for HPV, with an effective “screen and treat” program would provide a great benefit for all women in the developing world regardless of HPV exposure status [5]. With or without the VIA screening technique, there is no doubt that an effective “screen and treat” program must be established in resource poor areas to reduce the incidence of cervical cancer and the excessive number of cervical cancer deaths occurring in these regions of the world.

## 4. Conclusions

It is apparent that a current problem facing the developing world is a lack of cervical cancer screening. Women in resource poor areas often have higher exposure to cervical cancer risk factors including multiple sexual partners, poverty, multi-parity, tobacco use, malnutrition, and poor genital hygiene [4,5]. There is a distinct need for adequately funded, broad based “screen and treat” or “screen and refer” programs. Visual Inspection with Acetic acid is an inexpensive, effective, and versatile screening tool that can be performed in temporary or mobile clinics as well as more permanent health care sites. These characteristics make VIA a viable option for screening women in resource poor regions who do not have regular access to healthcare services.

Healthcare providers in developed nations have the ability to institute these programs by training doctors and nurses in resource poor areas. As this collaboration of Haiti Medishare and Emory School of medicine demonstrates, there are opportunities available to assist local health care providers in resource poor countries and demonstrate the most effective technique for VIA. By teaching local providers the skills needed to screen for cervical dysplasia, women with precancerous lesions can be identified in all clinical settings and then referred to sites offering appropriate follow-up care. The skills and materials required are minimal and the potential benefits are remarkable.
